# Gastrointestinal Stromal Tumors: Histopathological Spectrum, Molecular Subtypes, and Implications for Targeted Therapy

**DOI:** 10.7759/cureus.101180

**Published:** 2026-01-09

**Authors:** Hussein Qasim, Mohammad Abu Shugaer, Ahmad N Awawdeh, Tamara Dawaymeh, Karis Khattab, Musallam Al-oweiwi, Matteo Luigi Giuseppe Leoni, Giustino Varrassi

**Affiliations:** 1 Pathology and Laboratory Medicine, Jordan University of Science and Technology, Irbid, JOR; 2 Family Medicine, Jordan University of Science and Technology, Irbid, JOR; 3 Pathology and Laboratory Medicine, King Abdullah University Hospital, Irbid, JOR; 4 Faculty of Medicine, Jordan University of Science and Technology, Irbid, JOR; 5 Emergency Medicine, Marka Specialty Hospital, Amman, JOR; 6 Medical and Surgical Sciences and Translational Medicine, Sapienza University, Rome, ITA; 7 Pain Medicine, Fondazione Paolo Procacci, Rome, ITA

**Keywords:** gastrointestinal stromal tumor (gist), kit mutation, pdgfra mutation, sdh-deficient gist, tyrosine kinase inhibitors (tkis) therapy

## Abstract

Gastrointestinal stromal tumors (GISTs) are the most common mesenchymal neoplasms of the gastrointestinal tract and represent a paradigm of precision oncology in which histopathology and molecular profiling directly inform diagnosis, prognosis, and therapeutic strategy. Arising from the interstitial cells of Cajal or their precursors, GISTs display a wide morphological spectrum, including spindle cell, epithelioid, mixed, and rare histologic variants, often requiring immunohistochemical confirmation with KIT (CD117), DOG1, and CD34. Molecular characterization reveals a limited but clinically decisive range of driver mutations, most commonly in KIT and PDGFRA, with additional subsets involving SDH deficiency, NF1 alterations, and rare mutations such as BRAF, KRAS, or NTRK fusions. These molecular signatures underpin distinct biological behaviors, prognostic categories, and therapeutic sensitivities. Risk stratification incorporates tumor size, mitotic rate, anatomical location, and tumor rupture to predict recurrence and guide adjuvant therapy decisions. Targeted tyrosine kinase inhibitors (TKIs) have transformed GIST management, with imatinib as the foundational first-line therapy and subsequent agents, sunitinib, regorafenib, avapritinib, and ripretinib, addressing primary or secondary resistance driven by diverse mutational patterns. Nevertheless, challenges persist, including imatinib-resistant PDGFRA D842V mutations, SDH-deficient tumors lacking actionable kinase alterations, and the emergence of polyclonal secondary resistance due to heterogeneous KIT adenosine triphosphate (ATP)-binding pocket or activation loop mutations. Emerging therapeutic directions include next-generation kinase inhibitors, heat shock protein inhibitors, immunotherapy, metabolic and epigenetic targeting, and biomarker-driven individualized treatment strategies. This review synthesizes contemporary advances in the histopathological, molecular, and therapeutic landscape of GISTs, emphasizing an integrated diagnostic approach and highlighting ongoing efforts to overcome therapeutic resistance and optimize personalized care.

## Introduction and background

Gastrointestinal stromal tumors (GISTs) represent the most common mesenchymal neoplasms of the gastrointestinal tract and constitute a biologically and clinically distinctive group of tumors arising predominantly from the interstitial cells of Cajal or their precursors [1]. First recognized as a separate entity in the late 20th century with the advent of immunohistochemistry and molecular diagnostics, GISTs have since become a model disease in modern oncology, illustrating how precise histopathological characterization and molecular profiling can transform therapeutic outcomes [2]. Although relatively uncommon, with an estimated incidence of 10-15 cases per million population annually, GISTs account for most mesenchymal tumors in the gastrointestinal tract and present across a wide age range, with peak incidence in the sixth to seventh decades of life [2]. Most GISTs occur in the stomach (60-70%) and small intestine (20-30%), but they may arise anywhere along the gastrointestinal tract, including the esophagus, colon, rectum, and, less commonly, in extra-gastrointestinal locations such as the mesentery or omentum [3]. Clinically, their presentation varies widely depending on tumor size, location, and growth pattern [4]. Patients may be asymptomatic or present with nonspecific manifestations such as abdominal pain, early satiety, gastrointestinal bleeding, anemia, or an incidentally discovered mass [5]. Because of this heterogeneity, accurate diagnosis relies heavily on histopathological evaluation supplemented by immunohistochemistry and molecular assays [6]. The histopathological spectrum of GISTs is broad, encompassing spindle cell, epithelioid, and mixed morphologies, each of which may pose diagnostic challenges and overlap with other mesenchymal neoplasms [7]. Immunohistochemical expression of KIT (CD117) and DOG1 revolutionized diagnostic accuracy, yet a subset of GISTs remains negative for these markers, underscoring the importance of molecular testing [7]. Advances in genomic medicine have revealed that GISTs are driven by a relatively small number of recurrent mutations, most commonly in KIT and PDGFRA, but also in succinate dehydrogenase (SDH) complex genes, NF1, and, less frequently, BRAF, NTRK, or KRAS [8]. 

A key milestone in the management of GISTs was the introduction of tyrosine kinase inhibitors (TKIs), particularly imatinib, which dramatically improved outcomes in advanced disease and ushered in an era of precision oncology [9]. The clinical efficacy of TKIs is closely tied to the tumor’s mutational profile, with certain mutations predicting favorable responses while others, such as PDGFRA D842V or SDH deficiency, confer intrinsic resistance [10]. Subsequent development of second-, third-, and fourth-line agents, including sunitinib, regorafenib, avapritinib, and ripretinib, has expanded therapeutic options for patients with resistant or recurrent disease [11]. Despite these advances, significant challenges persist. Primary and secondary resistance to targeted therapy remains a major clinical obstacle, and rare molecular subtypes often lack effective treatment options [12]. Furthermore, evolving insights into the tumor microenvironment, epigenetic regulation, and metabolic reprogramming suggest that additional layers of complexity influence disease behavior and therapeutic response [13]. Accordingly, this review first outlines the histopathological spectrum of GISTs, then examines their molecular subtypes and prognostic stratification, and finally discusses the implications of these features for targeted therapy, resistance mechanisms, and emerging treatment strategies.

## Review

Methods

This narrative review was developed to synthesize current evidence on the histopathology, molecular characteristics, prognostic determinants, mechanisms of therapeutic resistance, and evolving treatment strategies in GISTs. A comprehensive literature search was conducted across PubMed, MEDLINE, Scopus, and Google Scholar, covering publications from 1995 to 2025. Search terms included “gastrointestinal stromal tumor”, “GIST”, “KIT mutation”, “PDGFRA mutation”, “SDH-deficient GIST”, “tyrosine kinase inhibitors”, “imatinib resistance”, “molecular subtypes”, and “targeted therapy”, combined using Boolean operators to broaden and refine the search strategy. Articles were selected for inclusion if they addressed key aspects relevant to this review, including histopathological patterns, molecular drivers, risk stratification systems, therapeutic responses, and mechanisms of resistance. Original research articles, clinical trials, expert guidelines, and high-quality review papers were prioritized, while non-English publications, abstracts without full text, and studies lacking relevance to the central themes were excluded. Reference lists of included studies were further screened to identify additional pertinent literature. Extracted information was synthesized qualitatively and organized into thematic domains reflecting the major components of GIST diagnosis and management: histopathological classification, molecular subtyping, prognostic modeling, targeted therapy, and emerging therapeutic approaches. Because this study relied exclusively on previously published literature and did not involve human subjects or patient data, institutional review board (IRB) approval was not required.

This article was conducted as a narrative review rather than a systematic review; therefore, Preferred Reporting Items for Systematic Reviews and Meta-Analyses (PRISMA) guidelines were not applied, and no formal study quality assessment or quantitative synthesis was performed.

Histopathological spectrum of GIST

GISTs demonstrate a broad range of gross pathological appearances that correlate with their site of origin, growth behavior, and underlying molecular subtype [14]. Tumor size varies from small incidental nodules to massive lesions exceeding 30 cm, although most symptomatic tumors measure between 5 cm and 15 cm at discovery [15]. Gastric GISTs often appear as well-circumscribed, lobulated masses arising from the muscularis propria, typically expanding outward in an exophytic fashion [16]. In contrast, small intestinal GISTs may exhibit a more infiltrative pattern with a higher likelihood of mucosal ulceration [17]. On cut surface, these tumors usually display a firm, fleshy, tan-white appearance with a whorled or trabeculated architecture [18]. Larger tumors commonly show areas of necrosis, hemorrhage, or cystic degeneration, findings that reflect rapid growth or impaired vascular supply [19]. Hemorrhage may be extensive, and cystic areas often develop as a result of tumor necrosis or myxoid change [20]. Tumor rupture, whether spontaneous or during surgical manipulation, is an important gross feature due to its strong association with increased recurrence risk [21]. Although most GISTs arise in the stomach or small intestine, occasional lesions occur in the colon, rectum, esophagus, or even in extra-gastrointestinal locations such as the mesentery and retroperitoneum, where they share similar gross characteristics [21,22].

Microscopically, GISTs encompass a spectrum of morphologies [23]. The spindle cell type, which constitutes approximately 70% of cases, is composed of uniform elongated cells arranged in interlacing fascicles or whorled bundles [24]. These cells have pale eosinophilic cytoplasm, tapered nuclei with fine chromatin, and may exhibit the classic perinuclear vacuolization seen particularly in gastric tumors [24]. Some spindle cell tumors also contain eosinophilic collagen globules known as skeinoid fibers, which are more frequently encountered in small bowel GISTs [24]. The epithelioid subtype, accounting for roughly 20-25% of cases, is characterized by round to polygonal cells with abundant eosinophilic or clear cytoplasm, arranged in nests, sheets, or syncytial aggregates [25]. Intracytoplasmic vacuoles and perinuclear clearing tend to be more prominent in this subtype, which is strongly associated with PDGFRA mutations, particularly the D842V variant [26]. Mixed-type GISTs display both spindle and epithelioid components, either in distinct regions or in a gradual transitional pattern, reflecting histologic and molecular heterogeneity [27]. Additionally, several uncommon variants enrich the morphological diversity of GISTs [28]. Sclerosing tumors exhibit dense hyalinized collagen, whereas palisaded tumors show nuclear palisading reminiscent of schwannomas despite retaining the immunophenotype of GIST [29]. Myxoid GISTs contain abundant mucoid stroma and are frequently linked to PDGFRA mutations [30]. Rarely, sarcomatoid transformation may occur, either de novo or as an acquired phenotype following prolonged TKI therapy, characterized by marked pleomorphism, high mitotic activity, and sometimes diminished KIT expression [31].

Immunohistochemistry plays a central role in the diagnosis of GISTs. KIT (CD117) remains the cornerstone marker, detected in approximately 95% of cases and showing strong cytoplasmic or membranous staining [32]. DOG1 has emerged as an equally essential marker due to its high sensitivity and specificity, particularly in KIT-negative tumors [33]. CD34 is positive in roughly 70-80% of GISTs, especially those of gastric origin [34]. Smooth muscle actin (SMA) may be expressed variably, more often in small intestinal tumors, while S100 is infrequently positive and may complicate differentiation from schwannomas [35]. Desmin is rarely expressed in GISTs, and its presence favors a true smooth muscle neoplasm over GIST [36]. In diagnostically challenging cases, particularly KIT-negative tumors that often harbor PDGFRA mutations, the combination of DOG1 positivity, CD34 expression, and supportive morphological findings helps confirm the diagnosis [37]. Molecular testing becomes especially important in these circumstances, as immunohistochemistry alone may be insufficient [38].

The differential diagnosis of GISTs includes several mesenchymal tumors with overlapping histologic features [39]. Leiomyomas and leiomyosarcomas may resemble spindle cell GISTs but typically demonstrate strong desmin and SMA reactivity and lack KIT and DOG1 expression [2]. Schwannomas may also mimic the spindle cell morphology of GISTs; however, they characteristically exhibit diffuse S100 positivity and a distinct peripheral lymphoid cuff, while being negative for KIT and DOG1 [40]. Inflammatory fibroid tumors can enter the differential diagnosis, particularly in cases with epithelioid morphology or PDGFRA mutations, but they show a prominent eosinophil-rich inflammatory infiltrate and remain negative for KIT and DOG1 [41]. Other smooth muscle tumors, such as myofibromas or fibromatoses, lack KIT/DOG1 expression and demonstrate distinct immunophenotypic patterns, including nuclear β-catenin staining in desmoid-type fibromatosis [42]. Careful correlation of morphology, immunohistochemistry, and molecular findings is therefore essential to distinguish GISTs from these entities and establish an accurate diagnosis.

Molecular subtypes of GIST

GISTs are defined by a relatively narrow but clinically significant spectrum of molecular alterations, the most common of which occur in the KIT gene [43]. Approximately 70-80% of GISTs harbor activating mutations in KIT, with exon 11 mutations being the most prevalent [44]. These mutations typically affect the juxtamembrane domain, leading to constitutive ligand-independent activation of the KIT receptor and downstream signaling pathways [45]. Exon 11 mutations are generally associated with favorable responses to imatinib therapy, although certain deletion mutations may predict more aggressive behavior [46]. Exon 9 mutations, more frequently identified in small bowel GISTs, affect the extracellular domain and are often associated with a more aggressive clinical course; they may require higher doses of imatinib to achieve optimal therapeutic responses [47]. Less commonly, mutations arise in exons 13 and 17, which involve the kinase domains and are typically associated with secondary resistance to TKIs [48]. The clinical significance of these mutations lies not only in their prognostic implications but also in their direct influence on therapeutic decision-making, as specific exon mutations predict varying degrees of sensitivity or resistance to available targeted agents [49].

PDGFRA-mutated GISTs constitute the second major molecular subgroup, accounting for approximately 10-15% of cases [50]. These tumors arise most commonly in the stomach and frequently exhibit epithelioid or mixed morphology [50,51]. Mutations occur predominantly in exons 12, 14, and 18, with exon 18 being the most frequent [43]. The D842V substitution in exon 18 is particularly noteworthy, as it confers strong resistance to imatinib and most conventional TKIs [52]. Tumors harboring this mutation often demonstrate bland epithelioid cytology, a myxoid stroma, and relatively indolent growth despite their inherent therapeutic resistance [53]. The advent of avapritinib has significantly improved treatment options for these patients, as it selectively targets PDGFRA D842V with high efficacy [54]. Other PDGFRA mutations, such as those in exon 12 or 14, are generally sensitive to imatinib, though the intensity of response varies [55]. Collectively, PDGFRA-mutant GISTs illustrate the importance of integrating molecular and morphologic features to guide diagnosis and management [55].

A distinct subgroup of GISTs is characterized by a deficiency in SDH, comprising approximately 5-7% of cases [56]. SDH-deficient GISTs typically arise in children, adolescents, and young adults, and they demonstrate unique clinical and pathological features [57]. These tumors are often multifocal, occur predominantly in the stomach, and display epithelioid cytology with a tendency toward a multinodular growth pattern [58]. SDH deficiency may occur in the context of the Carney triad, a nonhereditary disorder involving GISTs, pulmonary chondromas, and extra-adrenal paragangliomas, or Carney-Stratakis syndrome, an inherited condition defined by paraganglioma and GIST and associated with germline mutations in SDH subunits [59]. A hallmark diagnostic feature is the loss of SDHB immunostaining on immunohistochemistry, which indicates functional impairment of the SDH complex regardless of which subunit is mutated [59]. SDH-deficient GISTs are generally resistant to standard TKIs, emphasizing the need for alternative therapeutic strategies and highlighting a unique metabolic vulnerability as a potential target for future therapies [59].

Neurofibromatosis type 1 (NF1)-associated GISTs form another important molecular subgroup [60]. These tumors arise in patients with germline NF1 mutations and typically emerge in the context of other syndromic features such as café-au-lait macules, neurofibromas, or optic gliomas [61]. NF1-related GISTs usually originate in the small intestine and often present as multiple synchronous lesions [62]. Morphologically, they frequently manifest as spindle cell tumors with low mitotic activity [63]. Unlike KIT- or PDGFRA-mutated GISTs, NF1-associated tumors lack mutations in these genes and instead reflect activation of the RAS/MAPK pathway inherent to NF1 loss [64]. Although they may respond to TKIs in selected cases, these tumors generally show limited sensitivity to imatinib, necessitating alternative approaches to management [48].

In addition to these major groups, several rare molecular subtypes have been identified. BRAF-mutant GISTs, most commonly involving the V600E mutation, account for a very small subset of cases and typically arise in the small intestine [65]. These tumors do not harbor KIT or PDGFRA mutations but demonstrate activation of the MAPK pathway, raising the potential role of BRAF inhibitors in management [65]. KRAS mutations have also been reported, though they are exceedingly rare and often associated with resistance to imatinib [65]. Even more uncommon are GISTs with gene fusions involving NTRK or FGFR, which represent emerging molecular subsets of therapeutic interest, as TRK inhibitors or FGFR-targeted agents may be effective in these tumors [66]. Wild-type GISTs, defined as tumors lacking mutations in KIT, PDGFRA, SDH, NF1, and other canonical genes, constitute a heterogeneous group with evolving molecular classifications, including alterations in genes regulating chromatin remodeling, mitochondrial function, or cellular metabolism [67].

**Figure 1:**
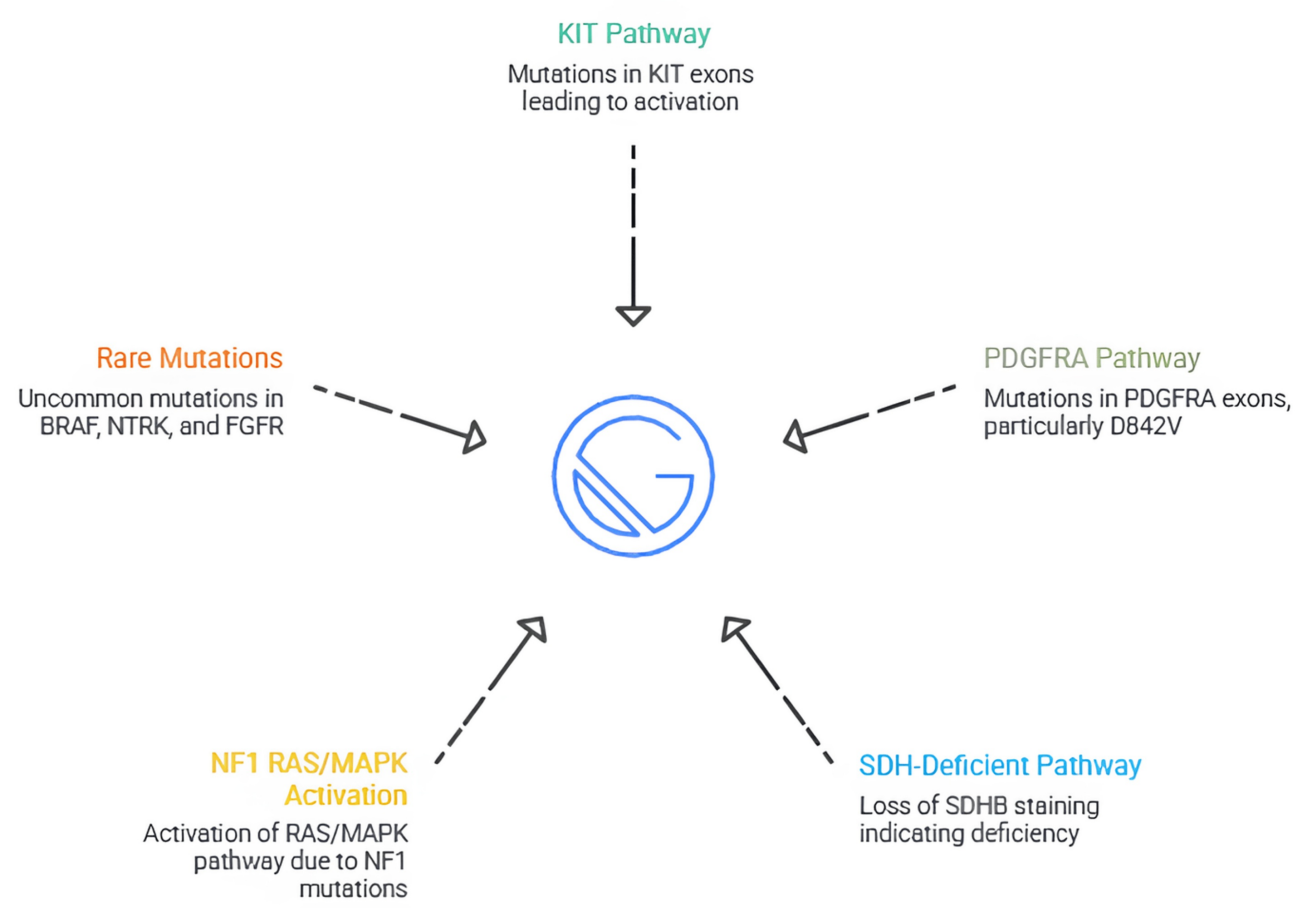
Summary of molecular subtypes of GIST. The figure is created by author Karis Khattab. GIST, gastrointestinal stromal tumor; NF1, neurofibromatosis type 1; SDH, succinate dehydrogenase

Given the profound therapeutic and prognostic implications of molecular classification, appropriate testing strategies are central to the management of GISTs [68]. Molecular testing is indicated at diagnosis for all newly resected or advanced tumors and is essential before initiating targeted therapy, as specific mutation types determine sensitivity to agents such as imatinib, sunitinib, regorafenib, avapritinib, or ripretinib [69]. Standard testing methods include targeted PCR-based assays for common KIT and PDGFRA exons, although next-generation sequencing (NGS) is increasingly preferred due to its broader coverage and ability to detect rare or complex mutations [8]. Immunohistochemical surrogates, such as SDHB staining for SDH-deficiency, help refine classification when sequencing results are absent or inconclusive [56]. Ultimately, integration of molecular data with histopathologic and clinical findings ensures accurate diagnosis and optimal individualized therapy. Figure 1 shows a summary of molecular subtypes of GIST.

Risk stratification and prognostic indicators

Accurate risk stratification is central to the management of GISTs, guiding decisions regarding surveillance, adjuvant therapy, and long-term follow-up [70]. The biological behavior of GISTs is heterogeneous, ranging from indolent, incidentally detected nodules to highly aggressive tumors with early metastatic potential [71]. Several established systems have been developed to predict recurrence risk, with the NIH/Fletcher and AFIP/Miettinen classifications being the most widely utilized [71]. The NIH/Fletcher criteria, introduced in 2002, categorize tumors into very low, low, intermediate, or high risk based on size and mitotic rate [72]. This model demonstrated the importance of tumor proliferative activity in forecasting recurrence but did not account for tumor location, which later emerged as an important determinant of outcome [52,72]. The AFIP/Miettinen criteria expanded upon this model by incorporating anatomical site, recognizing that gastric GISTs generally exhibit more favorable behavior than small intestinal counterparts of similar size and mitotic rate [14]. This refinement significantly improved risk stratification accuracy, particularly for intermediate-risk tumors [14]. More recent models have also incorporated additional parameters such as tumor rupture, either spontaneous or iatrogenic, which is now recognized as an independent and powerful predictor of peritoneal dissemination and recurrence, elevating such tumors to the highest risk category regardless of other features [73]. Collectively, these classification systems form the foundation of contemporary clinical decision-making.

Beyond traditional clinicopathologic indicators, molecular features have emerged as key prognostic determinants in GIST [74]. Mutation type strongly influences tumor behavior, response to therapy, and overall outcome [75]. KIT exon 11 mutations, particularly deletions involving codons 557-558, are associated with a higher risk of recurrence and metastasis compared with other mutation types, though they retain good responsiveness to imatinib [76]. Exon 9 mutations, more typical of small bowel tumors, portend a more aggressive clinical course and are less sensitive to standard-dose imatinib, often necessitating higher dosing strategies [77]. PDGFRA-mutant tumors, particularly those harboring the D842V mutation, display relative indolence but are inherently resistant to imatinib, making them poor candidates for conventional adjuvant therapy and more appropriate for targeted agents such as avapritinib [78]. Conversely, patients with exon 11 KIT mutations derive the greatest benefit from adjuvant imatinib, and current guidelines recommend three years of therapy for high-risk tumors based on robust data demonstrating improvements in recurrence-free and overall survival [79]. Thus, molecular profiling not only refines prognostication but also determines eligibility for adjuvant treatment and individualized therapy planning.

In parallel with advances in genomic characterization, growing attention has been directed toward the tumor microenvironment and emerging biomarkers that may further refine prognostication [80]. Immune infiltration patterns, including the presence of tumor-associated macrophages, T-cell subsets, and PD-L1 expression, have been linked to variable clinical outcomes, suggesting that GISTs may exhibit distinct immunologic phenotypes that influence response to therapy [81]. Epigenetic mechanisms, including promoter methylation signatures and chromatin remodeling, have been increasingly implicated in GIST biology, particularly in SDH-deficient tumors, where global DNA hypermethylation constitutes a defining feature [82]. These epigenetic alterations may contribute to tumorigenesis, metabolic dysregulation, and resistance to targeted therapies [83]. Additional biomarkers under investigation include circulating tumor DNA (ctDNA) for monitoring minimal residual disease, expression patterns of heat shock proteins (HSPs), and metabolic signatures that correlate with therapeutic response [84]. Although these markers remain primarily investigational, they hold considerable promise for improving individualized risk prediction and identifying patients who may benefit from emerging or combination therapeutic strategies.

Targeted therapy for GIST: current status

The advent of targeted TKIs has fundamentally transformed the management of GISTs, turning what was once a highly lethal disease into one of the most successful examples of precision oncology [9]. Therapeutic decisions are now closely aligned with the tumor's molecular profile, as specific mutations determine sensitivity or resistance to available agents [12]. First-line therapy is dominated by imatinib, while subsequent lines include sunitinib, regorafenib, avapritinib, and ripretinib, each addressing distinct mechanisms of primary or secondary resistance [85]. Despite major advancements, certain molecular subsets, particularly SDH-deficient and wild-type GISTs, continue to pose significant therapeutic challenges [69].

Imatinib remains the cornerstone of first-line therapy for advanced, metastatic, or unresectable GIST [86]. Its mechanism of action involves competitive inhibition of the adenosine triphosphate (ATP)-binding pocket of KIT and PDGFRA, effectively blocking downstream oncogenic signaling [85]. Clinical response to imatinib is highly mutation-dependent [85]. Tumors harboring KIT exon 11 mutations demonstrate the most favorable response rates and longest progression-free survival, whereas those with exon 9 mutations are less sensitive and may require higher dosing [87]. PDGFRA-mutated tumors, with the exception of non-D842V variants, typically respond well, while tumors carrying the PDGFRA D842V mutation exhibit marked resistance [26]. The mutation-specific effects of imatinib underscore the need for molecular testing in all newly diagnosed patients, as therapeutic outcomes cannot be reliably predicted based on morphology alone [88].

Sunitinib is the standard second-line therapy for patients who exhibit intolerance to or disease progression on imatinib. It targets a broader range of kinases, including KIT, PDGFRs, and VEGFRs, allowing it to overcome some mechanisms of imatinib resistance [89]. Secondary resistance often results from the emergence of additional KIT mutations in exons 13, 14, 17, or 18, which alter the kinase conformation and interfere with imatinib binding [90]. Sunitinib demonstrates activity against many of these ATP-binding pocket mutations, although its efficacy varies depending on the mutational pattern [91]. Clinical responses are commonly characterized by disease stabilization rather than dramatic tumor shrinkage, reflecting its role in managing disease heterogeneity and suppressing selectively resistant clones [92].

For patients who progress on sunitinib, regorafenib serves as the established third-line therapy [93]. Regorafenib inhibits multiple kinases implicated in GIST progression, including KIT, PDGFRA, FGFR, and angiogenic pathways [94]. Clinical trials have demonstrated meaningful progression-free survival benefits in heavily pretreated patients, emphasizing its utility even in tumors harboring diverse or complex resistance mutations [95]. Although objective response rates are modest, regorafenib provides durable disease control and is generally well tolerated with appropriate dose adjustments [96]. Its multitargeted mechanisms of action allow it to address multiple resistance pathways simultaneously, providing an important bridge toward newer therapeutic strategies [49].

Avapritinib represents a major advancement for patients with PDGFRA D842V-mutated GIST, a subgroup historically resistant to all conventional TKIs [97]. As a highly potent inhibitor that specifically targets the activation loop of PDGFRA, avapritinib achieves response rates far exceeding those of earlier agents [98]. It is now recognized as the standard of care for these tumors and has also demonstrated efficacy in some KIT-mutated GISTs, particularly those with activation loop mutations (exon 17) [99]. Avapritinib’s activity in advanced disease has reshaped treatment paradigms for this previously untreatable population, although careful monitoring is required due to neurocognitive adverse effects [100].

Ripretinib is a broad-spectrum switch-control TKI designed to inhibit a wide range of KIT and PDGFRA mutations, including both primary and secondary resistance mutations [101]. Its unique mechanism stabilizes the kinase in an inactive conformation, thereby preventing activation through multiple distinct mutational pathways [102]. Ripretinib is approved as fourth-line therapy and has demonstrated significant improvements in progression-free and overall survival compared with placebo [103]. Its ability to inhibit heterogeneous mutational subclones makes it particularly effective in later-line settings where resistance is multifaceted and evolving [103].

Therapeutic approaches to SDH-deficient and wild-type GISTs remain challenging, as these tumors typically lack actionable KIT or PDGFRA mutations and therefore show limited response to imatinib and other conventional TKIs [104]. SDH-deficient tumors, in particular, demonstrate global epigenetic dysregulation, metabolic rewiring, and unique transcriptional profiles that render kinase inhibition largely ineffective [105]. Investigational strategies for these molecular subsets include agents such as temozolomide, which may be effective due to hypermethylation-associated vulnerabilities, and inhibitors targeting IGF1R, which is frequently overexpressed in SDH-deficient GISTs [106]. Additional research is exploring the role of metabolic inhibitors, epigenetic therapies, and immunotherapy, although these remain in early developmental stages [107]. For now, management continues to rely on surgical intervention when feasible and participation in clinical trials for advanced disease. Table 1 presents a summary of medications used in the management of GISTs.

**Table 1:** Summary of used medications in the management of GISTs. GIST, gastrointestinal stromal tumor; ATP, adenosine triphosphate; SDH, succinate dehydrogenase; PFS, progression-free survival; OS, overall survival

Drug	Line of Therapy	Key Mechanism / Target	Mutation Sensitivity	Clinical Notes
Imatinib [108]	First-line [108]	Inhibits KIT and PDGFRA ATP-binding site [108]	Best response in KIT exon 11; reduced sensitivity in exon 9; no activity in PDGFRA D842V [108]	Dramatically improves survival; dose escalation for exon 9 tumors [108]
Sunitinib [109]	Second-line [109]	Inhibits KIT, PDGFRs, VEGFRs [109]	Effective in many secondary ATP-binding pocket mutations (exons 13/14) [109]	Useful after imatinib failure; stabilizes disease more than shrinking tumors [109]
Regorafenib [110]	Third-line [110]	Multikinase inhibitor (KIT, PDGFRA, FGFR, angiogenic pathways) [110]	Active against complex or mixed resistance profiles [110]	Improves PFS in heavily pretreated patients [110]
Avapritinib [111]	Special indication / first-line for PDGFRA D842V [111]	Potent inhibitor of PDGFRA activation loop [111]	Highly effective in PDGFRA D842V; also active in some exon 17 KIT mutations [111]	Breakthrough therapy for previously resistant subtype [111]
Ripretinib [101]	Fourth-line [101]	Switch-control inhibitor stabilizing inactive KIT/PDGFRA [101]	Broad activity against primary and secondary resistance mutations [101]	Improves OS/PFS; effective in mutationally heterogeneous disease [101]
Investigational agents (temozolomide, IGF1R inhibitors) [106]	Emerging therapies for SDH-deficient and wild-type GIST [106]	Target epigenetic, metabolic, or IGF1R pathways [106]	Useful in SDH-deficient tumors lacking KIT/PDGFRA mutations [106]	Consider for clinical trial enrollment [106]

Mechanisms of resistance to targeted therapy

Despite the transformative impact of TKIs on the management of GISTs, resistance to therapy remains a major clinical challenge [10]. Resistance may be present at the onset of treatment, known as primary resistance, or may develop after an initial period of disease control, termed secondary or acquired resistance [112]. These mechanisms are tightly linked to the underlying molecular profile of the tumor and significantly influence therapeutic outcomes, progression patterns, and survival [113]. Understanding the complex biology of resistance is essential for optimizing treatment strategies and guiding the development of next-generation targeted agents.

Primary resistance is defined as the failure to achieve a meaningful clinical response within the first six months of TKI therapy [113]. One of the most well-characterized causes of primary resistance is the PDGFRA D842V mutation, which occurs in the activation loop of the receptor and induces a conformation that prevents effective binding of imatinib and most other conventional TKIs [50]. Tumors harboring this mutation exhibit poor response rates despite often possessing relatively indolent histologic features [50]. The introduction of avapritinib, a highly selective inhibitor targeting this mutation, has significantly improved outcomes but does not entirely eliminate the challenge of resistance [114]. Another prominent cause of primary resistance occurs in SDH-deficient GISTs, which lack activating mutations in KIT or PDGFRA and instead display profound metabolic and epigenetic dysregulation [115]. Their oncogenic signaling is not driven by tyrosine kinase activation, rendering TKIs largely ineffective [115]. These tumors frequently arise in younger patients and may display slow-growing but relentlessly persistent disease, emphasizing the need for alternative therapeutic approaches beyond kinase inhibition [115].

Secondary resistance emerges after a period of initial clinical benefit and represents the most common mechanism of disease progression in metastatic GIST [12]. This resistance arises from the development of additional mutations in the KIT gene, often in different tumor subclones, creating what is referred to as polyclonal resistance [12]. These mutations typically occur in exons encoding the ATP-binding pocket (exons 13 and 14) or the activation loop (exons 17 and 18) [12]. Mutations in the ATP-binding pocket reduce the affinity of imatinib for KIT, while activation loop mutations stabilize the kinase in an active conformation that is resistant to drug inhibition [116]. The coexistence of multiple resistant subclones within the same tumor or across metastatic deposits is a hallmark of secondary resistance and contributes to the highly heterogeneous clinical responses observed with second- and third-line therapy [117]. Structural changes in the kinase domain further compound resistance by altering the three-dimensional configuration of the drug-binding site, thereby preventing effective inhibition even when TKIs are present at therapeutic levels [117].

**Figure 2:**
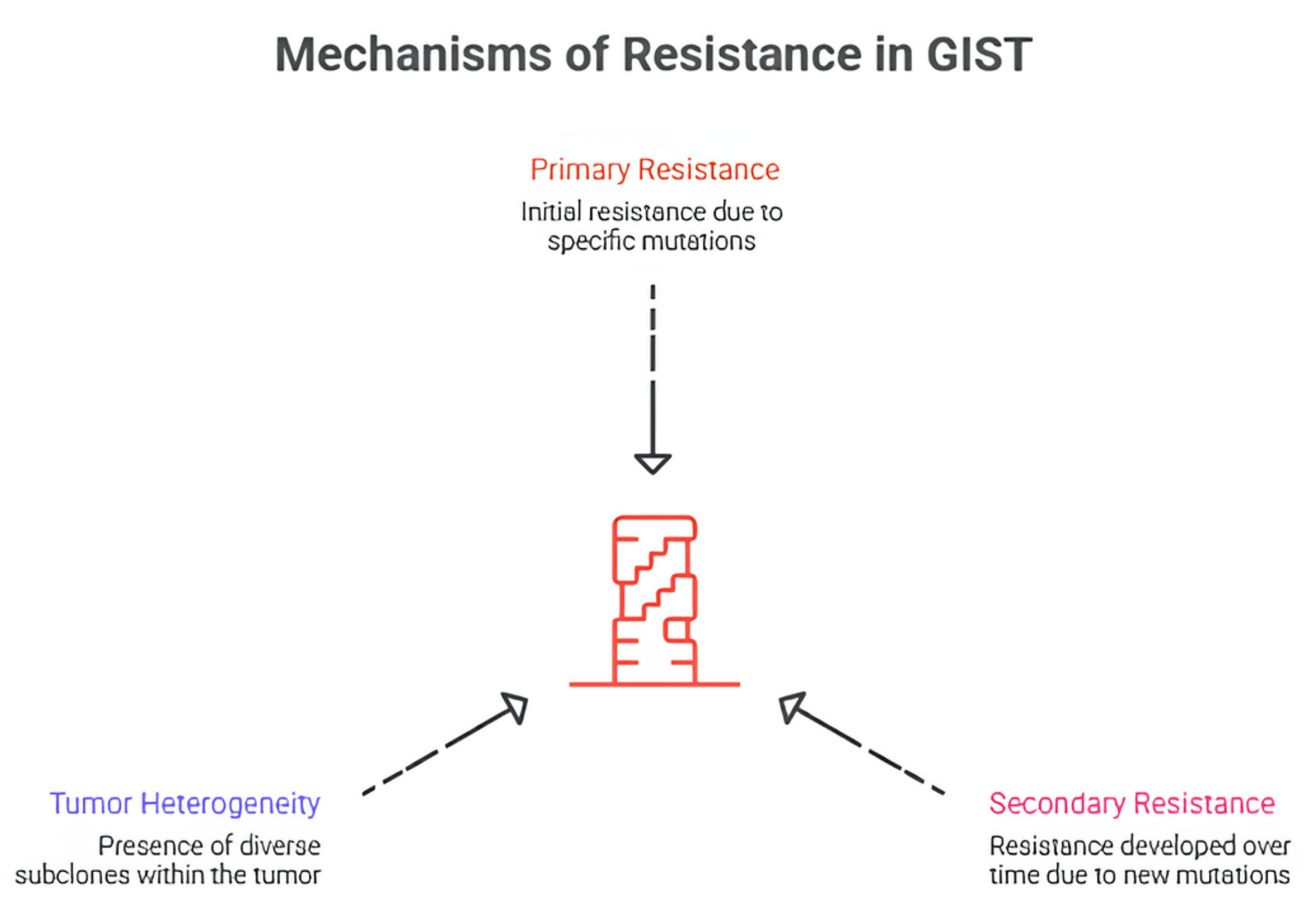
Summary of mechanisms of resistance in GIST. The figure is created by author Karis Khattab. GIST, gastrointestinal stromal tumor

Tumor heterogeneity plays a central role in shaping resistance patterns and is a critical driver of treatment failure in advanced GIST [118]. As selective pressure from TKIs preferentially suppresses sensitive clones, resistant clones accumulate and expand [119]. This evolutionary process leads to a mosaic of subpopulations with distinct mutational profiles, each with varying degrees of sensitivity to different TKIs [120]. This heterogeneity explains why sequential therapy often yields diminishing returns and why later-line agents, such as ripretinib, are designed with broad-spectrum inhibitory activity to simultaneously target multiple resistant clones [121]. Figure 2 shows a summary of mechanisms of resistance in GIST.

Emerging therapies and future directions

As insights into the molecular and cellular biology of GISTs continue to evolve, several emerging therapeutic strategies show promise for improving outcomes beyond current TKIs [99]. Novel kinase inhibitors are being developed to overcome resistant KIT and PDGFRA mutations, with next-generation agents designed to more effectively target activation loop mutations or bypass common resistance pathways [10]. HSP inhibitors represent another promising class, as they disrupt chaperone-mediated stabilization of mutant KIT, leading to degradation of the oncogenic protein [122]. Early studies have demonstrated potential synergy when HSP inhibitors are combined with TKIs, suggesting a role in mitigating resistance [123]. Immunotherapy is also being explored, particularly agents targeting the PD-1/PD-L1 axis, which may enhance antitumor immune responses in selected molecular subtypes [124]. Vaccine-based strategies and adoptive cell therapies remain in early experimental stages but highlight growing interest in leveraging immune modulation in GIST [125]. Combination therapy approaches, involving TKIs with metabolic inhibitors, epigenetic agents, or immune modulators, are under investigation to address tumor heterogeneity and prevent clonal escape [126]. Finally, advances in personalized and precision medicine, including the use of ctDNA, comprehensive genomic profiling, and adaptive treatment algorithms, are paving the way for more individualized therapeutic plans [127]. Collectively, these emerging strategies hold promise for improving disease control, overcoming resistance, and expanding treatment options for patients with challenging or refractory GIST subtypes.

Clinical implications and practical approach

The management of GISTs increasingly relies on the integration of histopathological assessment and molecular testing to achieve accurate diagnosis and guide clinical decision-making [128]. Histologic evaluation provides essential information regarding tumor morphology, mitotic activity, and risk stratification, while immunohistochemical markers such as KIT and DOG1 confirm the diagnosis in most cases [7]. However, molecular testing has become indispensable, as the specific mutation profile directly informs prognosis and predicts therapeutic response [129]. Tailoring therapy to mutation type is now a cornerstone of modern GIST management, with imatinib serving as first-line treatment for most KIT- and non-D842V PDGFRA-mutated tumors, while alternative agents such as avapritinib are required for PDGFRA D842V disease [52]. Similarly, the choice of second- or third-line therapy depends on the pattern of secondary resistance mutations that emerge during treatment, emphasizing the need for ongoing molecular evaluation when feasible [130]. Effective care for patients with GIST also requires a coordinated multidisciplinary approach [70]. Pathologists ensure accurate morphologic and molecular classification, surgeons guide decisions regarding resection or cytoreductive strategies, and medical oncologists determine appropriate systemic therapy and surveillance plans [131]. Collaboration across these specialties is essential for optimizing outcomes, especially in complex cases such as SDH-deficient tumors, recurrent disease, or tumors requiring neoadjuvant therapy [131].

## Conclusions

GISTs represent a paradigm of modern precision oncology, where the integration of histopathology and molecular profiling has dramatically advanced diagnostic accuracy and therapeutic outcomes. Key insights from decades of research have established the central role of KIT and PDGFRA mutations in driving tumor behavior and predicting response to targeted therapy, while also highlighting the unique biology of SDH-deficient, NF1-associated, and other rare molecular subtypes. Despite these advances, significant challenges persist, including primary and secondary resistance to TKIs, limited treatment options for wild-type and SDH-deficient tumors, and the complexity introduced by tumor heterogeneity and clonal evolution. As the field moves forward, continued innovations in molecular diagnostics, immunotherapy, next-generation kinase inhibition, and biomarker-driven precision medicine are expected to refine risk stratification and expand therapeutic options. The future of GIST management lies in increasingly individualized treatment strategies that leverage emerging technologies and multidisciplinary collaboration to overcome resistance, enhance disease control, and ultimately improve patient outcomes.
